# A new way forward? Examining the potential of quantitative analysis of IgE datasets

**DOI:** 10.1186/s13223-022-00717-8

**Published:** 2022-08-21

**Authors:** Felix King, Robert Kaczmarczyk, Alexander Zink, Tilo Biedermann, Knut Brockow

**Affiliations:** 1 School of Medicine, Department of Dermatology and Allergy, Technical University of Munich, Biedersteiner Str. 29, 80802 Munich, Germany; 2 Division of Dermatology and Venereology, Department of Medicine Solna, Karolinska Institutet, 17176 Stockholm, Sweden

**Keywords:** Allergy, Immunoglobulins, IgE sensitization, Aeroallergens, Food allergens

## Abstract

**Background:**

Allergies constitute an important public health problem, and epidemiological data is crucial to developing strategies for its prevention and therapy. Few population-based studies are available for data on allergies and sensitization. However, as these studies are expensive and time-consuming, novel approaches are searched for.

**Objectives:**

A large monocentric IgE dataset was used to analyse quantitative sensitization data in different age and gender groups and compared the results to available epidemiological data.

**Methods:**

A total of 14,370 patients who sought medical care at the Department for Dermatology and Allergology at the Technical University of Munich, Germany was analysed. Total IgE and sensitization measured in specific IgE levels to common food allergens and aeroallergens were compared between females and males, age groups, and the year of testing (2003–2021).

**Results:**

8283 females (57.6%) and 6087 males (42.4%) were tested. The average number of specific IgE tests per patient was 12.3 ± 11.4. Total IgE increased after birth with age and reached a peak between 4–6 years in males and 10–12 years in females. Males had higher specific IgE for all common aeroallergens (house dust mite, birch, mugwort and timothy grass pollen) and food allergens (milk protein, chicken egg white, peanut, wheat flour, cod) except for cat epithelia. Data closely reflected results of population-based studies in the literature.

**Conclusion:**

This study shows that, despite potential patient and test selection bias, the results of the quantitative IgE-dataset analysis closely reflect results of population-based data. Thus, as large cohorts can be examined with a minute amount of effort, this surrogate method appears promising to supplement epidemiology research.

**Supplementary Information:**

The online version contains supplementary material available at 10.1186/s13223-022-00717-8.

## Introduction

Allergic diseases constitute an important public health problem in Western societies [1, 2]. According to a representative survey conducted in the year 1998, about 40% of the respondents in Germany had already experienced allergies in their lifetime [3]. Especially in industrialized countries, a dramatic increase in prevalence was ‘traced’ in the last 50 years [4]. Allergies can lead to a significant reduction in quality of life [5], for example, by persistent nasal obstruction and eye watering (as in the case of tree pollen allergies) or by severe anaphylaxis (e.g. in peanut allergies). To develop adequate strategies, knowledge, prevention and therapy of allergic diseases, information on patients with allergic diseases is key.

Classic methods to generate this data include cohort and case-control studies as well as cross-sectional studies. These instruments have been successfully employed in the past to create much of the epidemiological backbone to allergy research [6]. However, these methods tend to be expensive, time-consuming and in the case of questionnaires often unreliable [7].

Specific IgE, together with skin prick testing, indicates sensitization, which is a measure and prerequisite for a potential IgE-mediated allergy. In the course of routine testing of allergy patients, large sets of IgE data are generated, which offer the potential to be pooled and analysed. We wanted to explore the potentials and pitfalls of quantitative IgE analysis and created a dataset by extracting the results of 14,370 allergy patients who had undergone IgE-testing. We analysed sensitizations over patient age and gender in order to elucidate possible trends to then appraise their validity by comparing the results with data from German health surveys [8, 9]. Moreover, sensitization levels between patients tested in the first and the second decade of the twenty-first century were compared to find potential sensitization shifts.

## Methods

### Patients

In this cross-sectional, retrospective, mono-centre study we used IgE sensitization data from 14,370 patients who sought medical care at the Department for Dermatology and Allergology, Technical University of Munich, Germany between January 2003 and February 2021. Most patients had symptoms suspected to be allergic (e.g., eczema, running nose, itchy eyes, anaphylactic reactions or skin rashes). Like similar studies in this field [10], the patients were grouped together. For this purpose, groups of three consecutive years of birth and all patients older than 81 years were bundled in order to increase the clarity and comparability of the groups.

### In-vitro total and specific IgE tests

Laboratory in-vitro tests from ImmunoCAP^®^ Phadia™, Laboratory Systems by Thermo Fisher Scientific were used to assess sensitization quantitatively measured in specific IgE antibodies. We analyzed the total IgE in kU/l and specific IgE levels in kUA/l for the following solutions: birch, mugwort and timothy grass pollen, house dust mite (Dermatophagoides pteronyssinus) and cat epithelia for aeroallergens as well as cow’s milk protein, chicken egg white, peanut, cod and wheat flour for food allergens. For statistical analysis, all values below the threshold [11] were treated as zeroes. *Dermatophagoides pteronyssinus* is referred to as *house dust mite* in our analysis.

### Statistical analysis

The data were analysed using Python 3.8.8 and the statistical libraries pandas 1.2.4, NumPy 1.20.1, and statsmodels 0.12.2 [12]. The mean and standard deviation or 95% confidence intervals were reported for continuous data, total number and the proportion in percentage are shown for categorical data. Differences between group means were assessed using one-way ANOVA and reported with p-values in addition to 95% confidence intervals. When categorical variables with only two groups were analysed, the estimated mean difference with 95% confidence intervals between both groups were shown.

## Results

### Demographic characteristics of the study population

The study population consisted of 8283 females (57.6%) and 6087 males (42.4%), altogether 12,834 adults (89.3%) and 1536 children (10.7%). The average age of the patients was 43.5 years (sd: 20.3) ranging between 0 and 106 years. The average number of specific IgE tests per patient was 12.3 (sd: 11.4, min: 0, max: 89), which did not differ between sex (p = 0.431) and age groups (p = 0.065). The top three tested allergens were timothy grass pollen (n = 6566, 45.7%), house dust mite (n = 6421, 44.7%) and birch pollen (n = 5935, 41.3%, Table 1).

**Table 1:** Comparison of total IgE and specific IgE values for several food and aeroallergens

Variable	n (%)	mean with 95% confidence interval			p-value	Sig
	Patients	Female (n = 8283, 57.6%)	Male (n = 6087, 42.4%)	Estimated difference		
Total IgE	10,099 (70.3)	225.9 ± 91.5	373.3 ± 92.1	+ 147.39 ± 31.41	< 0.001	***
Milk protein	5280 (36.7)	0.2 ± 9.7	0.6 ± 14.3	+ 0.44 ± 0.23	< 0.001	***
Chicken egg white	5234 (36.4)	0.3 ± 9.9	0.5 ± 13.1	+ 0.25 ± 0.21	0.02	*
Birch pollen	5935 (41.3)	8.1 ± 13.8	9.6 ± 13.9	+ 1.49 ± 1.08	0.007	**
Mugwort pollen	5821 (40.5)	1.0 ± 8.8	1.3 ± 8.9	+ 0.29 ± 0.25	0.021	*
Timothy grass pollen	6566 (45.7)	6.2 ± 13.7	9.4 ± 13.5	+ 3.23 ± 0.93	< 0.001	***
House dust mite	6421 (44.7)	3.9 ± 14.5	6.5 ± 14.6	+ 2.54 ± 0.82	< 0.001	***
Cat epithelia	5821 (40.5)	2.0 ± 12.9	2.4 ± 13.3	+ 0.35 ± 0.51	0.178	n.s
Peanut	3519 (24.5)	1.1 ± 11.9	1.9 ± 13.0	+ 0.72 ± 0.51	0.006	**
Wheat flour	5142 (35.8)	0.5 ± 8.3	0.8 ± 7.7	+ 0.29 ± 0.18	0.001	**
Cod	5155 (35.9)	0.1 ± 11.8	0.3 ± 13.2	+ 0.21 ± 0.17	0.011	*

For all tables and statistics: ns: p > 0.05, *: p < 0.05, **: p <0.01, ***: p < 0.001

### Total IgE values compared by age and gender

**Fig. 1:**
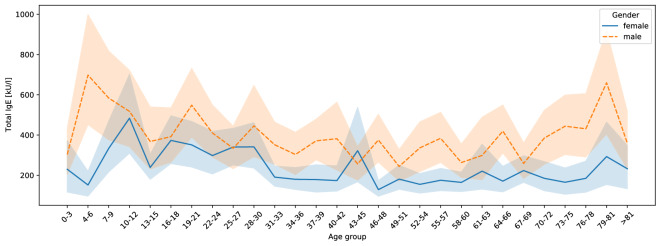
Comparison of total IgE by age and gender in 10,099 patients

The average total IgE, which was measured in more than 2/3 (n = 10,099, 70.3%) of all patients, was 292.3 kU/l (sd: 805.5). Total IgE in male patients (373.3 ± 92.1) was on average higher (+ 147.4 ± 31.4 kU/l, p < 0.001) than in female patients (225.9 ± 91.5, Table 1). Different age groups also showed significant differences for the total IgE (p < 0.001, Fig. 1, Additional file 1: Table S1). Especially high values were recorded for children in the age groups of 4–6 years (458.4 ± 82.6), 7–9 years (482.4 ± 70.8), 10–12 years (502.1 ± 75.6), 19–21 years (433.9 ± 88.8) and for the elderly between 79–81 years (480.9 ± 94.5). The lowest total IgE was seen for infants and children between 0–3 years (275.4 ± 80.7) and adults between 58–60 years (203.6 ± 66.2).

### Specific IgE values of food Allergens milk protein, chicken egg white, peanut, wheat flour and cod compared by age and gender

**Fig. 2:**
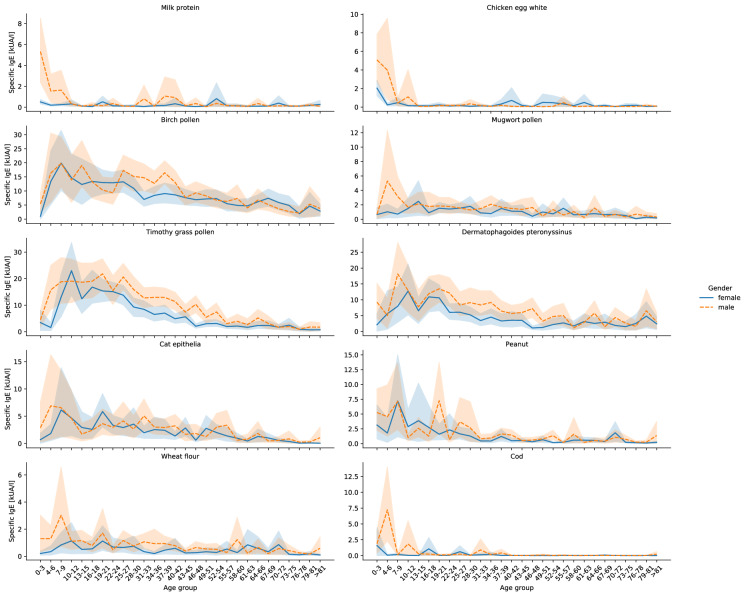
Line graphs showing the mean sensitization for ten different allergens in kUA/l with 95% confidence intervals for females and males in different age groups

All five analysed food allergens showed significant differences between age and gender groups. Again, males showed higher specific IgE values for chicken egg white (+ 0.2 ± 0.2 (p = 0.020), cow milk protein (+ 0.4 ± 0.2, p < 0.001), peanut (+ 0.7 ± 0.5, p = 0.006), wheat flour (+ 0.29 ± 0.18, p = 0.001) and cod (+ 0.21 ± 0.17, p = 0.011, Table 1). In terms of age groups, infants showed the highest sensitization rates to both chicken egg white (4.0 ± 12.9) and cow’s milk protein (3.5 ± 16.0), whereas the highest peanut, wheat flour and cod sensitization was seen in elementary school children between 7–9 years (7.2 ± 15.5, 2.0 ± 10.6 and 4.1 ± 14.3 respectively). From there on, the specific IgE values constantly decrease and reach a low plateau at the age of 13–15 years for chicken egg white (0.1 ± 1.6) and cow milk protein (0.1 ± 0.9). A low plateau for peanut sensitization occurs a little later at the age of 31–33 years (0.6 ± 4.9). Smaller sensitization peaks from 37 years on can be identified for cow milk protein, chicken egg white and peanut. The wheat flour sensitization remains stable with smaller peaks for patients older than 9 years, the sensitization to cod disappears from the age of 40 years onwards (Fig. 2, Additional file 1: Table S1).

A further distinction between age groups and gender reveals certain age groups with especially high differences for given allergens. Milk protein sensitization seems to be different between females and males only at the age group 0–3 years (males: + 4.82 ± 4.15), wheat flour at the age group 31–33 years (males: + 0.72 ± 0.56), while chicken egg white shows no differences in any age group. Cod sensitization differs in the age groups 4–6 years (males: + 7.16 ± 6.91) and 46–48 years (males: 0.04 ± 0.04) and peanut sensitization three distinct groups: 19–21 years (males: + 5.66 ± 5.10), 40–42 years (males: + 0.97 ± 0.94) and 52–54 years (males: + 1.18 ± 0.78, Additional file 2: Table S2).

### Specific IgE values of aeroallergens, birch, mugwort, timothy grass pollen, house dust mite and cat epithelia compared by age and gender

The aeroallergens show different characteristics compared to the food allergens. All analysed aeroallergens except for cat epithelia differed among sex groups (Table 1). Male patients showed on average a 1.5 ± 1.1 (p = 0.007) higher specific IgE for birch, 0.29 ± 0.25 (p = 0.021) for mugwort, 3.23 ± 0.93 for timothy grass pollen (p < 0.001) and 2.5 ± 0.8 (p < 0.001) for the house dust mite, while cat epithelia were the same for both females and males (0.3 ± 0.5, p = 0.178). Significant differences between age groups were seen for all analysed aeroallergens (p < 0.001, Fig. 2, Additional file 1: Table S1). They all start low after birth and while birch, house dust mite and cat epithelia reach their maximum in the age group between 7 and 9 years (19.9 ± 14.8, 13.9 ± 15.7 and 6.4 ± 16.4), mugwort pollen already shows the maximum between 4–6 years (3.5 ± 13.4) and timothy grass pollen a little bit later between 10–12 years (21.0 ± 14.6). From there on, the specific IgE almost steadily decreases for all aeroallergens. Both birch pollen and the house dust mite show another, smaller maximum in the elderly age group between 79 and 81 years (5.0 ± 15.8 and 5.7 ± 16.4). Regional minima are seen in the age group of 13–15 years for the house dust mite (7.2 ± 13.7) and cat epithelia (2.3 ± 10.7), before reaching a regional maximum in younger adults between 19–21 years (11.9 ± 14.1 and 4.8 ± 13.3 respectively, Additional file 1: Table S1). A big difference in sensitization in younger age groups between females and males was only shown for timothy grass, which shows a higher sensitization in males (+ 14.18 ± 10.22). The other aeroallergens show significant differences from the age group of 22 years onwards (Additional file 2: Table S2).

### Comparison of patients tested in first and second decade of the twenty-first century

A subgroup analysis for patients between 0 and 20 years showed no differences in the mean total IgE for patients tested before 2011 (403.02 ± 79.17) and after 2010 (383.97 ± 80.35, p = 0.67, Table 2). We found a reduction in sensitization to the aeroallergens timothy grass (−6.68 ± 3.85, p = 0.001) and house dust mite (−4.07 ± 3.21, p = 0.013) in the second decade. The food allergen sensitization for the cow’s milk protein (+ 1.46 ± 1.11, p = 0.01) and peanut (+ 3.04 ± 2.41, p = 0.014) increased. All other analysed allergens did not show a difference between the years of testing (Table 3).

**Table 2:** Comparison of the total IgE for young patients between 0 and 20 years tested in the year 2003–2010 versus 2011–2020

Age 0–20 years	Total IgE		
Year of test	n	mean with 95%-CI	Sig/p
2003–2010	686	403.02 ± 79.17	n.s
2011–2020	633	383.97 ± 80.35	0.669

**Table 3:** Comparison of specific IgE for given food and aeroallergens for young patients between 0 and 20 years tested in 2003 to 2010 versus 2011 to 2020

Aeroallergens	Birch pollen			Mugwort pollen			Timothy grass pollen			Dermatophagoides pteronyssinus			Cat epithelia		
Year of test	n	mean with 95% CI	p / Sig	n	mean with 95% CI	p/Sig	n	mean with 95% CI	p/Sig. /dif	n	mean with 95% CI	p/Sig./dif	n	mean with 95% CI	p/Sig
2003–2010	386	14.20 ± 14.90	0.346 n.s	394	2.03 ± 8.66	0.203 n.s	432	18.83 ± 14.02	0.001**	417	12.17 ± 14.64	0.013*	386	3.69 ± 12.59	0.89 n.s
2011–2020	358	12.28 ± 15.01		322	1.44 ± 9.81		431	12.16 ± 14.82	**−6.68 ± 3.85**	430	8.10 ± 14.76	**–4.07 ± 3.21**	334	3.83 ± 15.12	

Significant results shown as the mean difference within the 95% confidence intervals are marked in bold

## Discussion

Quantitative IgE analysis shows overall a clear match with the fluctuations of IgE findings over age already described in the literature. As in several other patient- and population-based studies, the quantitative IgE findings of this study show frequent sensitization to hen’s egg, cow’s milk, and peanut in the children studied [13]. Moreover, the results present young age and male gender as predictors of sensitizations [14].

Interestingly, with regard to the age of the sample population, we found an approximately bell-shaped distribution, which is interrupted, among other things, by a particularly high number of cases in 2–4-year-olds. Gellrich et al., who had performed a similar IgE serology study, had described a decline in the teenage years, which they had attributed to reduced participation in medical services [10]. Our sample had no such drop, which may be due to the fact that our highly specialized allergy centre is not a regular care facility but frequently visited by patients of all ages. This is corroborated by an average patient age of 43.5 years which closely reflects overall average age in Germany of 44.6 years [15]. Comparing patients whose serum was examined between 2003–2010 with those whose serum was examined between 2011 and 2020, our analysis shows a significant decrease in aeroallergen and increase in food sensitizations, with constant total IgE. This is interesting and may reflect a trend toward people seeking aid with possible food allergy which had previously not been that prominent in the media, whereas common allergic rhinoconjunctivitis is increasingly being treated by the general practitioner in Germany since reimbursement schemes have changed [16]. The finding still has to be confirmed by population-based data. It is noticeable that in the course of life there is a continuous decrease in total IgE, a phenomenon which is counted among the forms of immunosenescence [17, 18]. In 2005, Anja Mediaty and Karsten Neuber discovered a decrease in total and specific IgE in aging patients with asthma, allergic rhinitis and insect allergy but not in patients with atopic dermatitis [19].

As previously described in the literature, our data set also reflected epidemiological data with significantly higher rates of sensitization in the men than in the women [20–22]. Sun, Xiaxao, et al., examined a similarly sized sample of 15,534 Chinese patients for the presence of IgE sensitization. Gender comparison revealed significantly increased specific IgE positivities in the male group for almost all aero- and food allergens tested (including hen's egg, cow's milk, D. pteronyssinus, cat dander, dog dander, among others) with the exception of cowrie snail and mold mix compared to the female group. Although geographic and sociocultural differences undoubtedly influence allergens and allergen contact, both samples agree that males are more prone to sensitization than females [13]. That the male sex is more frequently affected by sensitization has been demonstrated in previous population-based studies [10, 23, 24]. While this discrepancy is unlikely to be explained by different allergen exposure, there are multiple hypotheses on the cause of male allergy preponderance ranging from different smoking habits [25] to differing dietary intakes of antioxidants [26].

Our results indicate significantly higher sensitizations in male sex to cow’s milk protein and chicken egg white, especially in infancy; a difference that dissipates around adolescence [23]. In a Swedish population-based birth cohort, Melén et al. followed over 4,000 subjects until 24 years of age. Their results also showed increased rates of cow's milk and chicken egg sensitization with a peak around 8 years of age, followed by a continuous decrease in specific IgE levels [27]. Again, male subjects were more frequently affected than females. As of now, only correlative relationships between allergy and sex could be established. The lack of comprehensive understanding of the root of this inter-sex discrepancy hampers the development of meaningful allergy prevention measures targeting males.

The earlier age peak for the above-mentioned food sensitizations in our German collective could be due to different diets or selection bias. According to this, young children in Germany could come into contact with hen's egg and cow's milk earlier or to higher amounts. Alternatively, patients may develop symptoms earlier than the general population. However, Skerven, et al. examined a Norwegian collective of infants in the first year of life. They were able to show that in this phase of life sensitization to chicken egg protein is particularly common, followed by cow's milk and peanut [14], which is in agreement with the results of our study as well as with those using American patient samples [28].

The Swedish researchers were able to demonstrate significantly higher rates of aeroallergen sensitization, esp. to timothy pollen, birch pollen, and cat epithelium, in the male subjects, which increased steadily until the final point of the study at 24 years of age. In fact, such a trend cannot be reconstructed from our data. In both sexes, the studied aeroallergens birch pollen, D. pteronyssinus, and cat epithelia show sensitization maximums earlier between 7 and 12 years of age with a subsequent slow gradual decrease in specific IgE levels over their lifetime. The present disparity might be due to a selection bias—in the Swedish birth collective, only new-born subjects were randomly selected, whereas our cross-sectional data are based on patients with predominantly specific allergological issues. Of course, lifestyle factors and differential allergen exposure could also play a role. As in the Swedish work, predominantly no gender-specific differences regarding possible food sensitization could be detected. Although some significant age-specific differences between the gender groups were found, it is difficult to determine their exact cause. Overall, however, it can be seen that the sensitizations investigated decrease over the lifetime irrespective of gender. In the case of food sensitizations, there is a pronounced decrease until young adulthood.

Using mono-centric Patient Data as done in this study has the limitation that samples are from patients whose serum was tested for the presence of IgE due to specific allergological problems or questions. Therefore, it has been unclear, if the results are applicable to the general population (selection bias). Furthermore, in many cases, allergen panels created specifically for the hospital were taken (e.g., aeroallergen or food allergen panel). This leads to the fact that only a small part of all possible sensitizations was measured in each patient. For rare allergens, sensitizations only in a small number of cases were determined. Interestingly, despite these limitations, our results correspond surprisingly well to data from birth cohorts and may offer new insights, which can be confirmed in population-based studies [9, 29].

On the other hand, mass quantitative IgE analysis has decisive advantages. First, population-based conclusions can be extracted from data about how sensitizations develop over long periods of time. In contrast to most studies, which mainly examine children for reasons of practicability, the present method makes it possible to easily examine a wide variety of age groups with a minute amount of effort involved.

In summary, the present data demonstrates that our patient-based data results are largely congruent with already existing population-based sensitization data and therefore appear to be a valid surrogate in allergy research. To fully exploit the potential of quantitative IgE analysis available for allergy research, further studies are needed. Among other things, it would be interesting to apply the same methodology to a dataset that includes diagnostics of an even broader spectrum of sensitization of each patient, e.g. as determined in Thermo Fisher's ISAC diagnostics [30]. Simultaneous acquisition of all technically detectable sensitizations would lead to both a higher hit rate and greater interindividual comparability. Pooling of data between centres and/or allergy laboratories would largely increase sample numbers and might even more closely reflect sensitizations present in the population. Large datasets collected in this way could subsequently be examined for possibly existing sensitization patterns and profiles using machine-learning, which could provide further exciting insights. In the future, those insights could be used to identify allergy clusters in the population. With concise data on age, sex and even regional distribution, allergy prevention measures could be precisely targeted for optimal effectiveness.

## Supplementary Information

**Additional file 1: Table S1.** Comparison of total and specific IgE for food and aeroallergens by age group.

**Additional file 2: Table S2.** Sex differences in sensitizations to food and aeroallergens by age group.

## Data Availability

The data that support the findings of this study are available from the corresponding author upon reasonable request. However, access to patient data which can be used to identify individuals cannot be granted.

## Abbreviations

D. pteronyssinus: Dermatophagoides pteronyssinus
IgE: Immunoglobulin E
