# Supplementary material for: A new way forward? Examining the potential of quantitative analysis of IgE datasets
Source: Allergy Asthma Clin Immunol. 2022 Aug 21;18:75. doi: 10.1186/s13223-022-00717-8 (PMC9394034; doi:10.1186/s13223-022-00717-8)
Supplement: Supplementary file 1 — Additional file 1: Table S1. Comparison of total and specific IgE for food and aeroallergens by age group. [file 13223_2022_717_MOESM1_ESM.docx]

|  | **Number of patients (%)** | **Total IgE** | **Milk protein** | **Chicken egg white** | **Birch pollen** | **Mugwort pollen** | **Timothy grass pollen** | **House dust mite** | **Cat epithelia** | **Peanut** | **Wheat flour** | **Cod** |
| --- | --- | --- | --- | --- | --- | --- | --- | --- | --- | --- | --- | --- |
| **Patients n (%)** | 14370 (100.0) | 10099 (70.3) | 5280 (36.7) | 5234 (36.4) | 5935 (41.3) | 5821 (40.5) | 6566 (45.7) | 6421 (44.7) | 5821 (40.5) | 3519 (24.5) | 5142 (35.8) | 5155 (35.9) |
| **Age group** |  |  |  |  |  |  |  |  |  |  |  |  |
| **0-3** | 354 (2.5) | 275.4 ± 80.7 | 3.5 ± 16.0 | 4.0 ± 12.9 | 4.2 ± 10.1 | 0.7 ± 5.1 | 4.1 ± 13.0 | 6.7 ± 16.5 | 2.2 ± 15.9 | 4.4 ± 15.0 | 0.9 ± 11.9 | 1.8 ± 11.2 |
| **4-6** | 197 (1.4) | 458.4 ± 82.6 | 0.9 ± 7.5 | 2.2 ± 14.9 | 15.0 ± 15.2 | 3.5 ± 13.4 | 9.6 ± 14.2 | 5.3 ± 13.8 | 4.8 ± 15.2 | 3.3 ± 13.2 | 0.9 ± 4.7 | 4.1 ± 14.3 |
| **7-9** | 226 (1.6) | 482.4 ± 70.8 | 1.0 ± 8.0 | 0.5 ± 5.1 | 19.9 ± 14.8 | 2.1 ± 8.9 | 16.3 ± 15.0 | 13.9 ± 15.7 | 6.4 ± 16.4 | 7.2 ± 15.5 | 2.0 ± 10.6 | 0.2 ± 3.2 |
| **10-12** | 241 (1.7) | 502.1 ± 75.6 | 0.3 ± 3.8 | 0.7 ± 12.4 | 14.3 ± 15.2 | 1.6 ± 6.4 | 21.0 ± 14.6 | 12.8 ± 15.0 | 4.6 ± 14.3 | 1.9 ± 8.1 | 1.1 ± 6.2 | 1.0 ± 17.9 |
| **13-15** | 269 (1.9) | 305.6 ± 67.5 | 0.1 ± 0.9 | 0.1 ± 1.6 | 16.0 ± 15.3 | 2.3 ± 9.5 | 15.7 ± 13.2 | 7.2 ± 13.7 | 2.3 ± 10.7 | 3.2 ± 13.5 | 0.9 ± 5.0 | 0.2 ± 7.4 |
| **16-18** | 380 (2.6) | 381.6 ± 75.4 | 0.1 ± 3.0 | 0.1 ± 2.7 | 13.3 ± 15.0 | 1.3 ± 8.4 | 17.7 ± 14.6 | 11.4 ± 14.7 | 2.5 ± 10.6 | 2.2 ± 14.1 | 0.7 ± 5.4 | 0.7 ± 14.1 |
| **19-21** | 507 (3.5) | 433.9 ± 88.8 | 0.4 ± 7.4 | 0.2 ± 4.7 | 11.8 ± 14.1 | 1.7 ± 7.2 | 18.1 ± 13.6 | 11.9 ± 14.1 | 4.8 ± 13.3 | 3.6 ± 14.9 | 1.4 ± 10.0 | 0.1 ± 2.5 |
| **22-24** | 603 (4.2) | 334.8 ± 91.3 | 0.2 ± 4.9 | 0.1 ± 2.8 | 11.7 ± 13.6 | 1.5 ± 6.8 | 15.2 ± 12.6 | 8.0 ± 12.8 | 3.2 ± 12.0 | 1.9 ± 12.8 | 0.6 ± 7.7 | 0.1 ± 3.2 |
| **25-27** | 687 (4.8) | 337.9 ± 79.8 | 0.1 ± 2.3 | 0.2 ± 3.6 | 14.9 ± 13.6 | 1.5 ± 7.0 | 16.7 ± 12.7 | 7.0 ± 13.7 | 3.4 ± 13.0 | 2.4 ± 11.9 | 0.8 ± 6.4 | 0.5 ± 11.7 |
| **28-30** | 651 (4.5) | 386.8 ± 104.8 | 0.1 ± 2.9 | 0.2 ± 5.6 | 12.8 ± 14.1 | 1.6 ± 8.8 | 12.3 ± 13.2 | 6.9 ± 13.3 | 3.1 ± 14.2 | 1.8 ± 14.2 | 0.7 ± 7.4 | 0.1 ± 1.9 |
| **31-33** | 679 (4.7) | 258.5 ± 71.6 | 0.4 ± 11.8 | 0.2 ± 4.1 | 10.3 ± 12.9 | 1.1 ± 7.4 | 10.3 ± 12.9 | 5.5 ± 13.3 | 3.3 ± 13.1 | 0.6 ± 4.9 | 0.6 ± 5.0 | 0.5 ± 16.0 |
| **34-36** | 672 (4.7) | 233.9 ± 78.5 | 0.1 ± 3.5 | 0.1 ± 2.1 | 10.5 ± 12.2 | 1.4 ± 8.7 | 9.6 ± 12.6 | 6.6 ± 13.9 | 2.8 ± 11.6 | 0.7 ± 6.0 | 0.5 ± 8.2 | 0.2 ± 7.8 |
| **37-39** | 738 (5.1) | 272.5 ± 84.9 | 0.6 ± 16.5 | 0.3 ± 7.8 | 12.9 ± 13.1 | 1.6 ± 9.0 | 10.1 ± 12.7 | 4.9 ± 14.2 | 2.7 ± 11.7 | 1.4 ± 7.8 | 0.7 ± 5.7 | 0.2 ± 8.2 |
| **40-42** | 684 (4.8) | 276.7 ± 117.7 | 0.6 ± 15.9 | 0.4 ± 17.2 | 10.7 ± 14.0 | 1.3 ± 7.2 | 8.1 ± 12.9 | 4.6 ± 14.0 | 2.2 ± 10.8 | 0.9 ± 5.3 | 0.7 ± 7.4 | 0.0 ± 1.6 |
| **43-45** | 730 (5.1) | 292.9 ± 150.6 | 0.1 ± 3.0 | 0.1 ± 4.5 | 7.7 ± 13.7 | 1.2 ± 11.1 | 6.4 ± 12.9 | 4.6 ± 14.6 | 2.3 ± 13.6 | 0.5 ± 5.1 | 0.3 ± 3.3 | 0.0 ± 1.4 |
| **46-48** | 779 (5.4) | 240.9 ± 89.7 | 0.2 ± 8.6 | 0.1 ± 1.4 | 8.0 ± 13.3 | 1.0 ± 10.5 | 6.0 ± 13.1 | 4.1 ± 15.0 | 1.2 ± 11.8 | 0.4 ± 3.7 | 0.4 ± 5.2 | 0.0 ± 1.8 |
| **49-51** | 693 (4.8) | 208.6 ± 73.7 | 0.1 ± 3.3 | 0.3 ± 13.9 | 7.7 ± 13.9 | 0.8 ± 10.4 | 4.1 ± 10.8 | 2.2 ± 13.7 | 2.1 ± 14.8 | 0.7 ± 6.1 | 0.4 ± 4.7 | 0.0 ± 4.8 |
| **52-54** | 672 (4.7) | 232.9 ± 85.6 | 0.6 ± 16.4 | 0.3 ± 10.8 | 7.0 ± 13.8 | 1.0 ± 9.0 | 5.1 ± 13.2 | 3.3 ± 15.2 | 2.4 ± 13.0 | 0.7 ± 6.0 | 0.4 ± 5.5 | 0.0 ± 1.5 |
| **55-57** | 652 (4.5) | 267.2 ± 87.0 | 0.1 ± 3.9 | 0.4 ± 10.4 | 5.9 ± 13.4 | 1.1 ± 11.2 | 2.4 ± 8.7 | 3.7 ± 15.6 | 2.3 ± 14.7 | 0.2 ± 3.5 | 0.4 ± 6.5 | 0.0 ± 3.2 |
| **58-60** | 618 (4.3) | 203.6 ± 66.2 | 0.1 ± 4.5 | 0.1 ± 4.8 | 5.8 ± 13.9 | 0.8 ± 7.5 | 2.8 ± 11.4 | 1.5 ± 12.6 | 0.8 ± 9.1 | 0.9 ± 13.0 | 0.6 ± 10.2 | 0.0 ± 1.4 |
| **61-63** | 559 (3.9) | 256.0 ± 130.1 | 0.1 ± 2.0 | 0.3 ± 12.2 | 4.4 ± 11.6 | 0.5 ± 6.2 | 2.1 ± 11.9 | 3.0 ± 14.1 | 0.6 ± 7.9 | 0.4 ± 5.0 | 0.6 ± 11.4 | 0.0 ± 1.8 |
| **64-66** | 617 (4.3) | 272.4 ± 87.2 | 0.2 ± 6.0 | 0.1 ± 2.2 | 6.4 ± 13.9 | 1.1 ± 11.1 | 3.5 ± 13.6 | 3.8 ± 16.3 | 1.5 ± 15.1 | 0.5 ± 5.3 | 0.6 ± 10.4 | 0.0 ± 1.9 |
| **67-69** | 536 (3.7) | 238.1 ± 70.8 | 0.1 ± 1.4 | 0.1 ± 3.4 | 6.4 ± 12.4 | 0.5 ± 5.1 | 2.7 ± 13.5 | 2.3 ± 15.2 | 0.8 ± 7.4 | 0.4 ± 4.4 | 0.3 ± 3.7 | 0.0 ± 3.8 |
| **70-72** | 471 (3.3) | 273.3 ± 84.7 | 0.3 ± 10.3 | 0.1 ± 1.2 | 4.9 ± 13.2 | 0.7 ± 8.4 | 1.7 ± 8.6 | 3.0 ± 15.1 | 0.6 ± 6.6 | 1.5 ± 9.8 | 0.7 ± 10.0 | 0.0 ± 1.2 |
| **73-75** | 396 (2.8) | 302.0 ± 85.9 | 0.1 ± 1.7 | 0.1 ± 3.8 | 3.8 ± 13.5 | 0.4 ± 5.5 | 2.2 ± 13.7 | 2.1 ± 14.8 | 0.6 ± 13.1 | 0.5 ± 4.8 | 0.3 ± 4.4 | 0.0 ± 0.7 |
| **76-78** | 304 (2.1) | 315.1 ± 84.3 | 0.1 ± 1.2 | 0.1 ± 2.2 | 2.0 ± 12.5 | 0.4 ± 10.4 | 0.9 ± 7.0 | 2.0 ± 12.7 | 0.2 ± 3.4 | 0.2 ± 2.0 | 0.2 ± 1.9 | 0.0 ± 0.4 |
| **79-81** | 197 (1.4) | 480.9 ± 94.5 | 0.2 ± 1.9 | 0.1 ± 1.8 | 5.0 ± 15.8 | 0.4 ± 4.3 | 1.2 ± 9.2 | 5.7 ± 16.4 | 0.2 ± 3.2 | 0.2 ± 2.6 | 0.2 ± 1.9 | 0.0 ± 0.7 |
| **>81** | 258 (1.8) | 295.1 ± 79.9 | 0.2 ± 4.6 | 0.1 ± 1.2 | 3.3 ± 12.0 | 0.3 ± 4.8 | 1.2 ± 9.8 | 2.7 ± 14.0 | 0.5 ± 14.4 | 0.7 ± 9.3 | 0.3 ± 6.5 | 0.1 ± 5.8 |
| **p** |  | <0.001 | <0.001 | <0.001 | <0.001 | <0.001 | <0.001 | <0.001 | <0.001 | <0.001 | <0.001 | <0.001 |
| **Sig.** |  | *** | *** | *** | *** | *** | *** | *** | *** | *** | *** | *** |
|  |  |  |  |  |  |  |  |  |  |  |  |  |
| **Table S1**: Comparison of total and specific IgE for food and aeroallergens by age group. | | | | | | |  |  |  |  |  |  |
