# Supplementary material for: A new way forward? Examining the potential of quantitative analysis of IgE datasets
Source: Allergy Asthma Clin Immunol. 2022 Aug 21;18:75. doi: 10.1186/s13223-022-00717-8 (PMC9394034; doi:10.1186/s13223-022-00717-8)
Supplement: Supplementary file 2 — Additional file 2: Table S2. Sex differences in sensitizations to food and aeroallergens by age group. [file 13223_2022_717_MOESM2_ESM.docx]

|  |  | **Total IgE** | | | **Milk protein** | | | **Chicken egg white** | | | **Birch pollen** | | | **Mugwort pollen** | | | **Timothy grass pollen** | | | **House dust mite** | | | **Cat epithelia** | | | **Peanut** | | | **Wheat flour** | | | **Cod** | | |
| --- | --- | --- | --- | --- | --- | --- | --- | --- | --- | --- | --- | --- | --- | --- | --- | --- | --- | --- | --- | --- | --- | --- | --- | --- | --- | --- | --- | --- | --- | --- | --- | --- | --- | --- |
| **Age group** | **Gender** | **n** | **mean** | **Sig.** | **n** | **mean** | **Sig.** | **n** | **mean** | **Sig.** | **n** | **mean** | **Sig.** | **n** | **mean** | **Sig.** | **n** | **mean** | **Sig.** | **n** | **mean** | **Sig.** | **n** | **mean** | **Sig.** | **n** | **mean** | **Sig.** | **n** | **mean** | **Sig.** | **n** | **mean** | **Sig.** |
| 0-3 | female | 91 | 229.99 ± 84.04 | n.s. | 83 | 0.51 ± 2.79 | * | 82 | 2.04 ± 5.26 | n.s. | 20 | 0.99 ± 7.78 | n.s. | 18 | 0.65 ± 4.48 | n.s. | 40 | 3.44 ± 13.12 | n.s. | 40 | 2.12 ± 14.32 | n.s. | 20 | 0.71 ± 5.94 | n.s. | 70 | 3.14 ± 13.67 | n.s. | 50 | 0.21 ± 2.31 | n.s. | 38 | 1.59 ± 13.62 | n.s. |
|  | male | 148 | 303.29 ± 79.23 |  | 139 | 5.33 ± 16.28 | **+4.82 ± 4.15** | 143 | 5.08 ± 13.98 |  | 50 | 5.42 ± 10.06 |  | 38 | 0.69 ± 5.35 |  | 72 | 4.50 ± 13.01 |  | 73 | 9.24 ± 16.54 |  | 41 | 2.90 ± 16.65 |  | 107 | 5.26 ± 15.49 |  | 85 | 1.30 ± 12.39 |  | 57 | 1.88 ± 9.68 |  |
| 4-6 | female | 50 | 151.73 ± 36.91 | ** | 39 | 0.20 ± 1.44 | n.s. | 39 | 0.24 ± 1.87 | n.s. | 25 | 13.37 ± 13.38 | n.s. | 19 | 1.03 ± 4.29 | n.s. | 31 | 1.57 ± 7.34 | ** | 30 | 5.61 ± 14.61 | n.s. | 21 | 1.83 ± 5.15 | n.s. | 37 | 1.79 ± 10.49 | n.s. | 29 | 0.34 ± 3.17 | n.s. | 31 | 0.07 ± 2.56 | * |
|  | male | 64 | 697.91 ± 84.08 | **+546.19 ± 323.00** | 45 | 1.55 ± 7.81 |  | 45 | 3.98 ± 15.15 |  | 31 | 16.33 ± 16.53 |  | 27 | 5.30 ± 14.15 |  | 40 | 15.75 ± 13.92 | **+14.18 ± 10.22** | 41 | 5.03 ± 13.39 |  | 29 | 6.89 ± 16.37 |  | 43 | 4.55 ± 13.94 |  | 35 | 1.30 ± 4.90 |  | 39 | 7.23 ± 14.04 | **+7.16 ± 6.91** |
| 7-9 | female | 49 | 333.70 ± 47.08 | n.s. | 40 | 0.25 ± 1.50 | n.s. | 41 | 0.48 ± 5.17 | n.s. | 38 | 19.94 ± 15.47 | n.s. | 35 | 0.72 ± 4.61 | n.s. | 41 | 13.06 ± 15.21 | n.s. | 38 | 7.95 ± 13.61 | n.s. | 36 | 6.15 ± 15.43 | n.s. | 38 | 7.21 ± 16.72 | n.s. | 36 | 0.83 ± 5.52 | n.s. | 37 | 0.17 ± 3.39 | n.s. |
|  | male | 72 | 583.58 ± 77.36 |  | 47 | 1.64 ± 8.38 |  | 45 | 0.44 ± 5.13 |  | 53 | 19.83 ± 14.38 |  | 46 | 3.14 ± 9.37 |  | 54 | 18.84 ± 14.88 |  | 53 | 18.16 ± 16.08 |  | 47 | 6.56 ± 17.17 |  | 44 | 7.15 ± 14.45 |  | 43 | 3.03 ± 11.35 |  | 40 | 0.13 ± 2.88 |  |
| 10-12 | female | 70 | 483.73 ± 73.78 | n.s. | 42 | 0.32 ± 4.34 | n.s. | 42 | 0.15 ± 1.21 | n.s. | 47 | 14.69 ± 14.79 | n.s. | 47 | 1.50 ± 7.40 | n.s. | 52 | 22.97 ± 14.96 | n.s. | 50 | 12.73 ± 15.92 | n.s. | 50 | 4.66 ± 13.81 | n.s. | 31 | 2.87 ± 9.23 | n.s. | 42 | 1.14 ± 7.64 | n.s. | 40 | 0.04 ± 1.12 | n.s. |
|  | male | 84 | 517.39 ± 77.36 |  | 48 | 0.31 ± 3.35 |  | 50 | 1.10 ± 13.14 |  | 48 | 13.91 ± 15.72 |  | 46 | 1.68 ± 5.41 |  | 55 | 19.04 ± 14.32 |  | 51 | 12.91 ± 14.27 |  | 47 | 4.59 ± 14.89 |  | 35 | 1.01 ± 3.60 |  | 49 | 1.10 ± 4.71 |  | 48 | 1.85 ± 18.06 |  |
| 13-15 | female | 93 | 239.04 ± 39.98 | n.s. | 44 | 0.12 ± 0.96 | n.s. | 44 | 0.14 ± 2.01 | n.s. | 52 | 12.31 ± 15.43 | n.s. | 51 | 2.49 ± 11.16 | n.s. | 58 | 12.39 ± 13.43 | n.s. | 55 | 6.52 ± 12.92 | n.s. | 52 | 2.94 ± 10.87 | n.s. | 33 | 3.87 ± 16.97 | n.s. | 45 | 0.50 ± 4.63 | n.s. | 44 | 0.01 ± 0.43 | n.s. |
|  | male | 99 | 368.09 ± 79.42 |  | 58 | 0.10 ± 0.91 |  | 56 | 0.08 ± 0.72 |  | 62 | 19.09 ± 15.14 |  | 65 | 2.13 ± 7.76 |  | 67 | 18.63 ± 13.02 |  | 68 | 7.79 ± 14.28 |  | 66 | 1.74 ± 10.49 |  | 34 | 2.59 ± 6.07 |  | 58 | 1.16 ± 5.05 |  | 58 | 0.29 ± 7.52 |  |
| 16-18 | female | 147 | 373.38 ± 74.19 | n.s. | 73 | 0.07 ± 0.66 | n.s. | 74 | 0.14 ± 3.23 | n.s. | 93 | 13.40 ± 14.94 | n.s. | 92 | 0.90 ± 5.56 | n.s. | 104 | 16.81 ± 15.23 | n.s. | 99 | 10.91 ± 15.26 | n.s. | 88 | 2.59 ± 11.01 | n.s. | 62 | 2.73 ± 15.43 | n.s. | 72 | 0.55 ± 6.19 | n.s. | 78 | 1.07 ± 15.26 | n.s. |
|  | male | 114 | 392.14 ± 77.18 |  | 61 | 0.21 ± 3.53 |  | 62 | 0.07 ± 0.76 |  | 71 | 13.26 ± 15.23 |  | 71 | 1.78 ± 9.83 |  | 76 | 19.02 ± 13.89 |  | 76 | 11.93 ± 14.11 |  | 69 | 2.43 ± 10.07 |  | 31 | 1.26 ± 5.38 |  | 62 | 0.79 ± 4.65 |  | 62 | 0.24 ± 3.57 |  |
| 19-21 | female | 206 | 351.18 ± 87.02 | n.s. | 119 | 0.52 ± 7.94 | n.s. | 116 | 0.23 ± 5.31 | n.s. | 124 | 12.98 ± 14.32 | n.s. | 135 | 1.53 ± 7.10 | n.s. | 147 | 15.35 ± 13.75 | n.s. | 137 | 10.61 ± 15.09 | n.s. | 120 | 5.85 ± 13.50 | n.s. | 87 | 1.59 ± 9.95 | * | 122 | 1.12 ± 10.10 | n.s. | 115 | 0.03 ± 0.96 | n.s. |
|  | male | 149 | 548.37 ± 89.68 |  | 73 | 0.14 ± 1.48 |  | 74 | 0.15 ± 2.43 |  | 100 | 10.34 ± 13.80 |  | 97 | 1.85 ± 7.28 |  | 110 | 21.80 ± 13.44 |  | 116 | 13.46 ± 13.08 |  | 103 | 3.63 ± 12.75 |  | 46 | 7.26 ± 16.35 | **+5.66 ± 5.10** | 84 | 1.71 ± 9.95 |  | 74 | 0.11 ± 2.90 |  |
| 22-24 | female | 265 | 298.07 ± 99.79 | n.s. | 141 | 0.13 ± 2.82 | n.s. | 135 | 0.12 ± 3.03 | n.s. | 163 | 12.91 ± 13.59 | n.s. | 164 | 1.42 ± 6.37 | n.s. | 179 | 15.10 ± 12.27 | n.s. | 172 | 6.04 ± 13.23 | * | 163 | 3.34 ± 12.85 | n.s. | 87 | 2.32 ± 13.35 | n.s. | 142 | 0.68 ± 8.66 | n.s. | 137 | 0.05 ± 2.96 | n.s. |
|  | male | 129 | 410.22 ± 76.66 |  | 60 | 0.35 ± 6.23 |  | 58 | 0.18 ± 2.32 |  | 80 | 9.32 ± 13.45 |  | 80 | 1.77 ± 7.38 |  | 86 | 15.50 ± 13.41 |  | 80 | 12.13 ± 12.08 | **+6.09 ± 4.87** | 76 | 2.97 ± 9.80 |  | 32 | 0.66 ± 3.20 |  | 67 | 0.47 ± 3.30 |  | 60 | 0.13 ± 3.42 |  |
| 25-27 | female | 252 | 340.40 ± 81.07 | n.s. | 135 | 0.12 ± 2.64 | n.s. | 134 | 0.17 ± 3.94 | n.s. | 155 | 13.25 ± 14.13 | n.s. | 162 | 1.54 ± 7.62 | n.s. | 169 | 13.76 ± 13.54 | * | 161 | 6.07 ± 13.86 | n.s. | 158 | 2.92 ± 11.32 | n.s. | 104 | 1.65 ± 8.34 | n.s. | 139 | 0.65 ± 7.05 | n.s. | 141 | 0.62 ± 12.75 | n.s. |
|  | male | 167 | 334.17 ± 78.09 |  | 86 | 0.11 ± 1.56 |  | 86 | 0.17 ± 3.00 |  | 106 | 17.30 ± 12.98 |  | 107 | 1.57 ± 5.93 |  | 125 | 20.64 ± 11.72 | **+6.88 ± 6.11** | 122 | 8.35 ± 13.64 |  | 107 | 4.14 ± 14.63 |  | 55 | 3.68 ± 14.18 |  | 89 | 1.16 ± 5.75 |  | 88 | 0.23 ± 4.74 |  |
| 28-30 | female | 221 | 341.28 ± 89.97 | n.s. | 107 | 0.12 ± 3.43 | n.s. | 109 | 0.09 ± 1.63 | n.s. | 124 | 10.95 ± 14.05 | n.s. | 133 | 1.79 ± 10.07 | n.s. | 133 | 9.31 ± 13.75 | * | 129 | 5.24 ± 11.59 | n.s. | 120 | 3.55 ± 14.93 | n.s. | 70 | 1.28 ± 8.16 | n.s. | 109 | 0.74 ± 8.49 | n.s. | 110 | 0.08 ± 2.02 | n.s. |
|  | male | 171 | 445.62 ± 117.71 |  | 80 | 0.08 ± 1.05 |  | 80 | 0.35 ± 6.37 |  | 99 | 15.18 ± 14.16 |  | 99 | 1.29 ± 5.62 |  | 107 | 15.91 ± 12.66 | **+6.60 ± 6.00** | 102 | 9.11 ± 14.37 |  | 94 | 2.64 ± 12.82 |  | 48 | 2.67 ± 17.29 |  | 82 | 0.76 ± 5.83 |  | 80 | 0.02 ± 0.73 |  |
| 31-33 | female | 250 | 190.96 ± 59.79 | ** | 119 | 0.07 ± 1.46 | n.s. | 116 | 0.14 ± 3.96 | n.s. | 148 | 6.98 ± 11.45 | ** | 146 | 0.88 ± 6.97 | n.s. | 159 | 8.43 ± 13.31 | n.s. | 147 | 3.43 ± 12.21 | * | 135 | 1.98 ± 9.74 | n.s. | 88 | 0.46 ± 3.83 | n.s. | 120 | 0.34 ± 4.14 | * | 121 | 0.13 ± 7.15 | n.s. |
|  | male | 181 | 351.74 ± 78.50 | **+160.78 ± 111.83** | 91 | 0.81 ± 12.37 |  | 89 | 0.17 ± 4.34 |  | 113 | 14.73 ± 13.37 | **+7.75 ± 5.11** | 105 | 1.47 ± 7.69 |  | 120 | 12.72 ± 12.36 |  | 109 | 8.37 ± 13.72 | **+4.93 ± 3.94** | 106 | 5.07 ± 14.34 |  | 56 | 0.82 ± 5.66 |  | 88 | 1.07 ± 5.23 | **+0.72 ± 0.56** | 94 | 0.89 ± 17.24 |  |
| 34-36 | female | 259 | 180.03 ± 66.05 | * | 134 | 0.14 ± 4.15 | n.s. | 128 | 0.09 ± 2.54 | n.s. | 134 | 8.43 ± 12.54 | n.s. | 134 | 0.79 ± 8.95 | n.s. | 148 | 6.51 ± 12.99 | ** | 148 | 4.54 ± 14.17 | * | 136 | 2.55 ± 13.83 | n.s. | 83 | 0.44 ± 6.83 | n.s. | 127 | 0.20 ± 3.39 | n.s. | 134 | 0.20 ± 9.20 | n.s. |
|  | male | 201 | 303.35 ± 86.28 | **+123.33 ± 112.65** | 93 | 0.10 ± 1.47 |  | 94 | 0.08 ± 1.25 |  | 123 | 12.72 ± 11.81 |  | 118 | 2.08 ± 8.52 |  | 134 | 12.95 ± 12.14 | **+6.44 ± 4.61** | 123 | 9.15 ± 13.54 | **+4.61 ± 4.35** | 115 | 3.01 ± 8.89 |  | 76 | 0.95 ± 5.56 |  | 90 | 0.93 ± 9.18 |  | 93 | 0.17 ± 4.72 |  |
| 37-39 | female | 259 | 178.80 ± 83.11 | ** | 128 | 0.16 ± 3.93 | n.s. | 126 | 0.33 ± 9.38 | n.s. | 136 | 9.09 ± 13.46 | * | 137 | 1.49 ± 10.91 | n.s. | 153 | 7.02 ± 12.87 | * | 145 | 3.31 ± 13.61 | n.s. | 137 | 2.42 ± 13.13 | n.s. | 89 | 1.21 ± 8.80 | n.s. | 127 | 0.45 ± 5.95 | n.s. | 128 | 0.03 ± 1.47 | n.s. |
|  | male | 246 | 371.10 ± 84.81 | **+192.30 ± 124.09** | 113 | 1.07 ± 17.82 |  | 112 | 0.18 ± 2.31 |  | 142 | 16.49 ± 12.68 | **+7.40 ± 5.60** | 132 | 1.68 ± 6.73 |  | 165 | 12.93 ± 12.44 | **+5.90 ± 4.50** | 154 | 6.45 ± 14.39 |  | 141 | 2.93 ± 10.51 |  | 77 | 1.64 ± 6.78 |  | 113 | 0.93 ± 5.51 |  | 112 | 0.42 ± 8.56 |  |
| 40-42 | female | 244 | 173.71 ± 74.89 | * | 132 | 0.33 ± 10.41 | n.s. | 130 | 0.72 ± 18.01 | n.s. | 150 | 8.62 ± 14.13 | n.s. | 153 | 1.12 ± 8.02 | n.s. | 158 | 4.88 ± 11.08 | ** | 154 | 3.54 ± 16.48 | n.s. | 150 | 1.38 ± 7.68 | n.s. | 80 | 0.49 ± 4.62 | * | 131 | 0.59 ± 9.37 | n.s. | 130 | 0.04 ± 1.80 | n.s. |
|  | male | 239 | 381.78 ± 132.34 | **+208.07 ± 177.86** | 109 | 0.93 ± 17.81 |  | 109 | 0.08 ± 2.18 |  | 136 | 13.05 ± 13.78 |  | 131 | 1.50 ± 6.48 |  | 153 | 11.37 ± 13.35 | **+6.49 ± 4.11** | 145 | 5.69 ± 12.09 |  | 129 | 3.24 ± 11.95 |  | 68 | 1.45 ± 5.49 | **+0.97 ± 0.84** | 109 | 0.78 ± 5.13 |  | 109 | 0.03 ± 1.39 |  |
| 43-45 | female | 289 | 322.07 ± 182.49 | n.s. | 147 | 0.12 ± 2.68 | n.s. | 145 | 0.18 ± 5.02 | n.s. | 167 | 7.62 ± 13.51 | n.s. | 170 | 1.09 ± 9.74 | n.s. | 187 | 5.63 ± 13.57 | n.s. | 181 | 3.50 ± 15.06 | n.s. | 171 | 2.84 ± 13.23 | n.s. | 100 | 0.53 ± 4.44 | n.s. | 146 | 0.24 ± 3.55 | n.s. | 145 | 0.02 ± 1.62 | n.s. |
|  | male | 232 | 256.60 ± 78.45 |  | 124 | 0.15 ± 3.33 |  | 124 | 0.05 ± 1.15 |  | 143 | 7.71 ± 13.94 |  | 140 | 1.37 ± 12.23 |  | 158 | 7.39 ± 12.22 |  | 146 | 5.98 ± 14.19 |  | 139 | 1.68 ± 14.46 |  | 71 | 0.52 ± 5.90 |  | 128 | 0.39 ± 3.05 |  | 126 | 0.01 ± 0.48 |  |
| 46-48 | female | 302 | 128.86 ± 58.81 | *** | 148 | 0.06 ± 1.00 | n.s. | 145 | 0.06 ± 0.95 | n.s. | 178 | 6.90 ± 11.59 | n.s. | 164 | 0.42 ± 6.53 | * | 182 | 2.05 ± 8.54 | *** | 179 | 1.10 ± 7.78 | *** | 168 | 0.61 ± 6.03 | n.s. | 97 | 0.31 ± 2.35 | n.s. | 146 | 0.26 ± 4.12 | n.s. | 145 | 0.01 ± 0.78 | * |
|  | male | 252 | 375.27 ± 98.04 | **+246.42 ± 117.34** | 127 | 0.36 ± 9.46 |  | 130 | 0.09 ± 1.63 |  | 151 | 9.31 ± 14.60 |  | 147 | 1.64 ± 11.32 | **+1.22 ± 1.19** | 165 | 10.33 ± 13.45 | **+8.27 ± 3.35** | 170 | 7.20 ± 15.57 | **+6.10 ± 3.20** | 150 | 1.83 ± 13.28 |  | 74 | 0.60 ± 4.33 |  | 130 | 0.65 ± 5.54 |  | 130 | 0.05 ± 1.89 | **+0.04 ± 0.04** |
| 49-51 | female | 261 | 180.87 ± 73.02 | n.s. | 138 | 0.12 ± 3.72 | n.s. | 136 | 0.51 ± 14.35 | n.s. | 150 | 7.17 ± 13.62 | n.s. | 150 | 1.01 ± 11.89 | n.s. | 163 | 3.00 ± 9.36 | n.s. | 160 | 1.27 ± 14.25 | n.s. | 154 | 2.78 ± 16.48 | n.s. | 94 | 0.66 ± 7.05 | n.s. | 136 | 0.33 ± 5.02 | n.s. | 141 | 0.01 ± 0.92 | n.s. |
|  | male | 206 | 243.79 ± 74.33 |  | 100 | 0.05 ± 0.77 |  | 98 | 0.04 ± 1.22 |  | 129 | 8.26 ± 14.18 |  | 125 | 0.46 ± 4.22 |  | 142 | 5.43 ± 11.58 |  | 136 | 3.25 ± 13.41 |  | 127 | 1.26 ± 8.86 |  | 68 | 0.78 ± 4.75 |  | 98 | 0.54 ± 4.38 |  | 100 | 0.09 ± 5.21 |  |
| 52-54 | female | 268 | 155.00 ± 68.08 | ** | 133 | 0.82 ± 18.71 | n.s. | 132 | 0.47 ± 11.43 | n.s. | 161 | 7.29 ± 13.79 | n.s. | 155 | 0.75 ± 6.01 | n.s. | 169 | 3.13 ± 9.55 | * | 163 | 2.18 ± 15.01 | n.s. | 151 | 2.02 ± 12.17 | n.s. | 91 | 0.17 ± 2.05 | ** | 129 | 0.28 ± 5.46 | n.s. | 135 | 0.01 ± 0.92 | n.s. |
|  | male | 202 | 336.33 ± 93.82 | **+181.33 ± 121.13** | 103 | 0.37 ± 5.95 |  | 102 | 0.08 ± 1.24 |  | 121 | 6.72 ± 13.77 |  | 116 | 1.35 ± 10.66 |  | 138 | 7.45 ± 14.56 | **+4.32 ± 3.39** | 132 | 4.71 ± 15.32 |  | 116 | 2.99 ± 13.77 |  | 73 | 1.35 ± 6.27 | **+1.18 ± 0.78** | 98 | 0.51 ± 5.48 |  | 105 | 0.03 ± 1.62 |  |
| 55-57 | female | 249 | 175.36 ± 71.82 | ** | 133 | 0.14 ± 4.43 | n.s. | 131 | 0.34 ± 11.40 | n.s. | 143 | 5.50 ± 14.54 | n.s. | 141 | 1.52 ± 12.54 | n.s. | 161 | 1.95 ± 7.90 | n.s. | 160 | 2.71 ± 13.96 | n.s. | 142 | 1.40 ± 14.50 | n.s. | 92 | 0.23 ± 4.16 | n.s. | 127 | 0.53 ± 7.58 | n.s. | 133 | 0.01 ± 1.11 | n.s. |
|  | male | 196 | 383.84 ± 93.54 | **+208.49 ± 134.90** | 102 | 0.16 ± 3.10 |  | 103 | 0.48 ± 9.39 |  | 117 | 6.28 ± 12.01 |  | 115 | 0.59 ± 5.12 |  | 129 | 3.06 ± 9.28 |  | 124 | 5.00 ± 16.69 |  | 114 | 3.36 ± 14.74 |  | 59 | 0.19 ± 1.71 |  | 105 | 0.28 ± 2.67 |  | 104 | 0.06 ± 3.61 |  |
| 58-60 | female | 264 | 164.72 ± 63.76 | * | 138 | 0.14 ± 4.77 | n.s. | 136 | 0.15 ± 5.25 | n.s. | 148 | 4.88 ± 11.85 | n.s. | 148 | 0.62 ± 6.71 | n.s. | 162 | 2.13 ± 10.17 | n.s. | 168 | 1.74 ± 14.09 | n.s. | 154 | 0.97 ± 9.75 | n.s. | 95 | 0.59 ± 9.23 | n.s. | 132 | 0.27 ± 3.15 | n.s. | 138 | 0.03 ± 1.53 | n.s. |
|  | male | 171 | 263.53 ± 67.93 | **+98.81 ± 92.56** | 82 | 0.12 ± 4.00 |  | 77 | 0.05 ± 1.14 |  | 91 | 7.34 ± 15.81 |  | 91 | 1.05 ± 8.14 |  | 104 | 3.93 ± 12.32 |  | 103 | 1.14 ± 8.01 |  | 94 | 0.61 ± 7.28 |  | 50 | 1.60 ± 15.13 |  | 78 | 1.23 ± 11.75 |  | 79 | 0.01 ± 0.49 |  |
| 61-63 | female | 231 | 220.58 ± 125.07 | n.s. | 119 | 0.10 ± 1.89 | n.s. | 117 | 0.50 ± 12.84 | n.s. | 138 | 4.72 ± 12.49 | n.s. | 131 | 0.68 ± 6.35 | n.s. | 146 | 1.66 ± 11.69 | n.s. | 145 | 3.13 ± 15.30 | n.s. | 134 | 0.46 ± 5.84 | n.s. | 85 | 0.58 ± 5.39 | n.s. | 122 | 0.85 ± 12.22 | n.s. | 120 | 0.02 ± 2.08 | n.s. |
|  | male | 192 | 298.71 ± 134.47 |  | 81 | 0.09 ± 2.08 |  | 81 | 0.08 ± 2.76 |  | 103 | 3.99 ± 10.06 |  | 98 | 0.25 ± 5.72 |  | 119 | 2.65 ± 12.15 |  | 115 | 2.90 ± 12.33 |  | 101 | 0.80 ± 9.11 |  | 56 | 0.21 ± 2.93 |  | 79 | 0.23 ± 3.33 |  | 85 | 0.01 ± 0.74 |  |
| 64-66 | female | 295 | 171.02 ± 86.47 | *** | 167 | 0.10 ± 3.48 | n.s. | 166 | 0.10 ± 2.48 | n.s. | 185 | 6.17 ± 13.64 | n.s. | 183 | 0.78 ± 9.26 | n.s. | 182 | 2.34 ± 12.05 | n.s. | 189 | 2.54 ± 15.07 | n.s. | 185 | 1.29 ± 14.71 | n.s. | 92 | 0.53 ± 5.46 | n.s. | 159 | 0.59 ± 12.34 | n.s. | 168 | 0.03 ± 2.25 | n.s. |
|  | male | 203 | 419.66 ± 85.84 | **+248.64 ± 129.84** | 98 | 0.36 ± 6.83 |  | 98 | 0.10 ± 1.72 |  | 109 | 6.79 ± 14.46 |  | 105 | 1.56 ± 12.47 |  | 123 | 5.20 ± 14.51 |  | 126 | 5.79 ± 17.01 |  | 107 | 1.84 ± 15.69 |  | 63 | 0.52 ± 5.03 |  | 94 | 0.67 ± 6.45 |  | 100 | 0.04 ± 1.54 |  |
| 67-69 | female | 246 | 223.52 ± 70.74 | n.s. | 128 | 0.12 ± 1.22 | n.s. | 128 | 0.17 ± 3.70 | n.s. | 137 | 7.42 ± 13.53 | n.s. | 134 | 0.64 ± 5.66 | n.s. | 153 | 2.36 ± 12.91 | n.s. | 152 | 2.93 ± 16.81 | n.s. | 136 | 1.02 ± 8.15 | n.s. | 79 | 0.35 ± 3.57 | n.s. | 126 | 0.33 ± 4.09 | n.s. | 128 | 0.07 ± 4.13 | n.s. |
|  | male | 173 | 258.80 ± 71.07 |  | 91 | 0.10 ± 1.58 |  | 90 | 0.06 ± 1.21 |  | 106 | 5.16 ± 9.93 |  | 104 | 0.40 ± 3.66 |  | 115 | 3.11 ± 14.18 |  | 115 | 1.52 ± 10.10 |  | 101 | 0.46 ± 4.66 |  | 50 | 0.40 ± 5.41 |  | 90 | 0.20 ± 2.51 |  | 92 | 0.02 ± 1.60 |  |
| 70-72 | female | 215 | 184.94 ± 73.26 | ** | 115 | 0.40 ± 11.54 | n.s. | 114 | 0.05 ± 0.92 | n.s. | 134 | 5.90 ± 13.70 | n.s. | 129 | 0.65 ± 9.53 | n.s. | 140 | 1.70 ± 9.27 | n.s. | 146 | 1.90 ± 12.21 | n.s. | 130 | 0.56 ± 5.79 | n.s. | 68 | 1.83 ± 10.68 | n.s. | 116 | 0.86 ± 10.43 | n.s. | 117 | 0.02 ± 1.13 | n.s. |
|  | male | 170 | 385.03 ± 89.97 | **+200.08 ± 143.03** | 98 | 0.13 ± 1.99 |  | 97 | 0.09 ± 1.40 |  | 111 | 3.69 ± 11.99 |  | 107 | 0.67 ± 6.81 |  | 118 | 1.67 ± 7.83 |  | 116 | 4.39 ± 16.46 |  | 110 | 0.57 ± 7.41 |  | 55 | 1.08 ± 7.65 |  | 97 | 0.57 ± 9.19 |  | 98 | 0.03 ± 1.22 |  |
| 73-75 | female | 164 | 165.39 ± 63.43 | ** | 83 | 0.10 ± 1.68 | n.s. | 83 | 0.17 ± 4.35 | n.s. | 106 | 4.88 ± 13.92 | n.s. | 102 | 0.52 ± 5.90 | n.s. | 113 | 2.47 ± 15.41 | n.s. | 110 | 1.52 ± 11.99 | n.s. | 102 | 0.37 ± 5.11 | n.s. | 62 | 0.21 ± 2.50 | n.s. | 86 | 0.15 ± 2.10 | n.s. | 83 | 0.01 ± 0.53 | n.s. |
|  | male | 158 | 443.86 ± 91.48 | **+278.47 ± 164.51** | 84 | 0.13 ± 1.80 |  | 84 | 0.06 ± 0.84 |  | 101 | 2.67 ± 12.71 |  | 100 | 0.34 ± 4.77 |  | 118 | 1.92 ± 11.19 |  | 118 | 2.61 ± 16.16 |  | 100 | 0.85 ± 15.33 |  | 57 | 0.72 ± 5.28 |  | 81 | 0.42 ± 4.99 |  | 85 | 0.02 ± 0.70 |  |
| 76-78 | female | 115 | 184.88 ± 61.42 | * | 58 | 0.11 ± 1.44 | n.s. | 58 | 0.19 ± 2.48 | n.s. | 68 | 1.91 ± 14.48 | n.s. | 68 | 0.08 ± 1.92 | n.s. | 73 | 0.89 ± 7.13 | n.s. | 74 | 2.53 ± 13.57 | n.s. | 66 | 0.07 ± 2.33 | n.s. | 33 | 0.15 ± 2.10 | n.s. | 59 | 0.11 ± 1.74 | n.s. | 59 | 0.01 ± 0.36 | n.s. |
|  | male | 129 | 431.20 ± 90.32 | **+246.32 ± 190.69** | 59 | 0.10 ± 0.91 |  | 58 | 0.05 ± 0.62 |  | 75 | 2.16 ± 10.83 |  | 72 | 0.70 ± 10.84 |  | 88 | 0.96 ± 6.85 |  | 87 | 1.64 ± 11.47 |  | 73 | 0.25 ± 3.60 |  | 35 | 0.26 ± 1.87 |  | 58 | 0.25 ± 1.90 |  | 57 | 0.01 ± 0.48 |  |
| 79-81 | female | 79 | 292.93 ± 83.72 | * | 40 | 0.18 ± 2.03 | n.s. | 39 | 0.07 ± 0.79 | n.s. | 47 | 4.63 ± 15.24 | n.s. | 49 | 0.29 ± 4.17 | n.s. | 53 | 0.71 ± 4.81 | n.s. | 55 | 4.84 ± 15.38 | n.s. | 48 | 0.10 ± 2.20 | n.s. | 24 | 0.10 ± 1.27 | n.s. | 37 | 0.18 ± 2.36 | n.s. | 39 | 0.01 ± 0.68 | n.s. |
|  | male | 83 | 659.82 ± 97.14 | **+366.88 ± 324.25** | 38 | 0.23 ± 1.78 |  | 38 | 0.17 ± 2.13 |  | 51 | 5.32 ± 16.37 |  | 48 | 0.49 ± 4.38 |  | 55 | 1.75 ± 10.43 |  | 60 | 6.55 ± 17.17 |  | 51 | 0.30 ± 3.44 |  | 21 | 0.24 ± 3.10 |  | 37 | 0.16 ± 1.10 |  | 38 | 0.03 ± 0.65 |  |
| >81 | female | 119 | 232.62 ± 77.41 | n.s. | 60 | 0.27 ± 5.17 | n.s. | 58 | 0.10 ± 1.24 | n.s. | 73 | 2.85 ± 11.27 | n.s. | 72 | 0.19 ± 2.54 | n.s. | 88 | 0.73 ± 5.50 | n.s. | 86 | 2.42 ± 13.35 | n.s. | 72 | 0.06 ± 1.61 | n.s. | 36 | 0.22 ± 2.47 | n.s. | 57 | 0.10 ± 1.31 | n.s. | 60 | 0.01 ± 0.43 | n.s. |
|  | male | 115 | 359.82 ± 81.24 |  | 47 | 0.10 ± 1.11 |  | 47 | 0.09 ± 1.11 |  | 69 | 3.78 ± 12.54 |  | 69 | 0.32 ± 5.73 |  | 86 | 1.72 ± 11.18 |  | 87 | 3.05 ± 14.62 |  | 69 | 1.06 ± 14.75 |  | 28 | 1.36 ± 10.18 |  | 46 | 0.59 ± 7.07 |  | 47 | 0.22 ± 5.96 |  |
|  |  |  |  |  |  |  |  |  |  |  |  |  |  |  |  |  |  |  |  |  |  |  |  |  |  |  |  |  |  |  |  |  |  |  |
| **Table S2**: Sex differences in sensitizations to food and aeroallergens by age group | | | | | |  |  |  |  |  |  |  |  |  |  |  |  |  |  |  |  |  |  |  |  |  |  |  |  |  |  |  |  |  |
